# The Metagenome of *Utricularia gibba*'s Traps: Into the Microbial Input to a Carnivorous Plant

**DOI:** 10.1371/journal.pone.0148979

**Published:** 2016-02-09

**Authors:** Luis David Alcaraz, Shamayim Martínez-Sánchez, Ignacio Torres, Enrique Ibarra-Laclette, Luis Herrera-Estrella

**Affiliations:** 1 Laboratorio Nacional de Ciencias de la Sostenibilidad, Departamento de Ecología de la Biodiversidad, Instituto de Ecología, Universidad Nacional Autónoma de México, AP 70–275, 04510, Ciudad Universitaria, Ciudad de México, México; 2 Instituto de Investigaciones en Ecosistemas y Sustentabilidad, Universidad Nacional Autónoma de México, Antigua Carretera a Pátzcuaro 8701, 58190, Morelia, Michoacán, México; 3 Red de Estudios Moleculares Avanzados, Instituto de Ecología, A.C, 91070, Carretera antigua a Coatepec 351, El Haya Xalapa, Veracruz, México; 4 Laboratorio Nacional de Genómica para la Biodiversidad (LANGEBIO), Centro de Investigación y de Estudios Avanzados del Instituto Politécnico Nacional (CINVESTAV), Km 9.6 Carretera Irapuato-León, 36821, Irapuato, Guanajuato, México; University of Illinois at Urbana-Champaign, UNITED STATES

## Abstract

The genome and transcriptome sequences of the aquatic, rootless, and carnivorous plant *Utricularia gibba* L. (Lentibulariaceae), were recently determined. Traps are necessary for *U*. *gibba* because they help the plant to survive in nutrient-deprived environments. The *U*. *gibba*'s traps (Ugt) are specialized structures that have been proposed to selectively filter microbial inhabitants. To determine whether the traps indeed have a microbiome that differs, in composition or abundance, from the microbiome in the surrounding environment, we used whole-genome shotgun (WGS) metagenomics to describe both the taxonomic and functional diversity of the Ugt microbiome. We collected *U*. *gibba* plants from their natural habitat and directly sequenced the metagenome of the Ugt microbiome and its surrounding water. The total predicted number of species in the Ugt was more than 1,100. Using pan-genome fragment recruitment analysis, we were able to identify to the species level of some key Ugt players, such as *Pseudomonas monteilii*. Functional analysis of the Ugt metagenome suggests that the trap microbiome plays an important role in nutrient scavenging and assimilation while complementing the hydrolytic functions of the plant.

## Introduction

Plant-microbe interactions have historically been studied by means of culture techniques. Culture dependent techniques, and directed experiments (*i*.*e*. reporter genes) have identified nitrogen-fixing, and plant growth-promoting bacteria, mychorrhizae fungi, pathogens, parasites, and protozoa that have a direct influence on plant health and development [1]. More than 30 years of research on plant-microbe interactions have expanded our horizons from the pathogen to beneficial microbe dichotomy to the current understand of microbes as a complex community associated with the plant, even being part of plant's extended phenotype [2]. Recently, next-generation sequencing platforms have allowed the study of microbiomes, which has yielded information about a large portion of plant-associated bacteria that cannot be easily cultivated *in vitro*. Several recent reports have focused on the study of the microbial diversity in the rhizosphere and endosphere of several plants species, including *Arabidopsis thaliana*, rice, and maize [3–7], however, they were performed primarily using 16S rRNA deep sequencing. Although 16S rRNA gene alignment and comparison is the gold standard, microbiome studies using this technology provide only proxy information about the potential functions harbored by the microbial communities. Hence, describing the taxonomic and functional diversity of microbiomes requires the use of whole-genome shotgun (WGS) metagenomics to complement these types of studies. The genome and transcriptome of the aquatic, rootless, carnivorous plant *Utricularia gibba* have recently been published [8,9]. The *U*. *gibba*'s small genome of 82 Mb hosts around 28,500 predicted coding genes (CDS), and several whole genome duplications. *U*. *gibba* has specialized trap structures that supplement the plant with Nitrogen (N), and Phosphorus (P) by digesting the preys. The nutrients generated by carnivory are relevant for the plant as it is generally inhabiting oligotrophic environments with limited supplies of N and P [8,10,11]. The environment inside the traps of *U*. *gibba* may be analogous to a rhizosphere in which the plant exchanges organic exudates derived from photosynthesis with its microbial community, with in turn enhances the capacity of the plant root to scavenge N and P from the soil. Due to the nature of the trap and its biophysical mechanisms to catch prey, the trap environment has very low levels of oxygen and high production of reactive oxygen species (ROS) [8,9]. Before the transcriptomic of the *U*. *gibba's* trap was determined, it was expected that the microbial community within the trap was solely responsible for prey digestion. However, transcriptome analysis of the trap revealed that several genes encoding hydrolytic enzymes are actively expressed in this organ, suggesting a coordinated functional expression of the host and the trap microbiome genomes for prey digestion [10,12,13]. There is a growing interest on the study of carnivorous plant microbiomes, there are recent publications involving metatranscriptomic and amplicon sequencing and analysis of the microbiomes for Genlisea genus and several sister species of Utricularia: *U*. *vulgaris*, *U*. *reflexa*, *U*. *australis*, and *U*. *intermedia*. Both *Genlisea* and *Utricularia* works rely on cultivated plants under laboratory conditions [14,15].

Recently, a two-step selection model for root microbiota differentiation was proposed [2]. The two step model takes into account the following considerations: edaphic or in our particular case water-substrate factors that determine the microbial diversity in the substrate; then the plant rhizodeposits organic compounds originated from photosynthesis, and plant's cell wall that could trigger chemotaxis mechanisms in the microbes promoting differential growth; finally, the plant genotype could select the community that it allows to growth within the plant.

In this work, we collected *U*. *gibba* plants from a natural habitat and sequenced a composite WGS metagenomes using approximately 400 *U*. *gibba*'s traps (Ugt) and the surrounding water and sediments (Ugm). Here, we show a thorough analysis of the microbial taxonomic and functional diversity in the Ugt and Ugm. We used the transcriptomic and genomic data available for *U*. *gibba* to verify if there is a functional complementation between the plant and its trap microbiome. The overall diversity is also compared with several related, publicly available metagenomes, including tropical soils, plant-associated samples, and water samples.

## Materials and Methods

### Sampling

A total of 13 *Utricularia gibba* L. (Lentibulariaceae) specimens were collected in the Trans-Mexican volcanic belt in shallow fresh waters, next to a dam near the locality of Umecuaro, Municipality of Morelia, in the Mexican state of Michoacan (19.53 N, -101.25 W; elev. 2191 masl; collection date: 07-12-2013 13:14:00 UTC -6; water temperature 18°C). The plant was identified in the field by its flower. Surrounding water and substrate (~5 cm depth) were also collected in sterile 50 ml tubes. All the samples were thoroughly collected and placed in individual sterile containers and immediately frozen in liquid nitrogen. No special permissions were required for the sampling location, and the land owner allowed us to carry on the collect. *U*. *gibba* is a pan-tropical distributed species and abundant in wide geographical area, so it is not an endangered nor protected species it is ranked as Least Concern (LC) in the Red List of the International Union for Conservation of Nature (IUCN).

### DNA extraction and library preparation

DNA from each subsample (water, substrate and traps) was isolated using an extraction buffer with 100 mM Tris- HCI pH 8.0; 50 mM EDTA pH 8.0; 500 mM NaCl 1.2% β-mercaptoethanol, 2% SDS and 10% PVP. Equal concentrations (10 μg) of environment DNA (water and substrate) were mixed to make a composite genetic pool representing the total DNA composition for *U*. *gibba*’s surroundings, we did not get enough DNA from water to perform WGS sequencing so we mixed it with the muddy sediment. The *U*. *gibba*'s surroundings worked as a baseline and reference to compare the environment and *U*. *gibba*'s microbiome. We considered that the shallow waters (>30 cm), and the underlying sediments are a single system. The goal to study the mixed water-sediment metagenome is that phenomena like chemotaxis to plant's exudates could drive the establishment of a distinctive bacterial community along with substrate dependent community structure, as it has been suggested [2]. The *U*. *gibba*'s traps were separated individually from the plant and pooled to extract the DNA; approximately 400 traps (»100 mg) were used. DNA purity and concentration were analyzed using a NanoDrop spectrophotometer. Libraries were constructed and sequenced at the Genomic Services laboratory, Unidad de Genomica Avanzada (UGA, formerly LANGEBIO-CINVESTAV), Mexico, using the MiSeq™ platform according to the manufacturer's instructions (Illumina, San Diego, CA).

### Sequence processing

We sequenced the samples using Illumina's MiSeq™ with paired ends in a 2x250 bp configuration for the *U*. *gibba*'s traps and environment. Pair-end reads were merged using PANDASEQ [16]. Quality checks and trimming (Q>30, 98% of the sequence) were performed using the FASTX_Toolkit (http://hannonlab.cshl.edu/fastx_toolkit/). Using Bowtie we performed an initial alignment and screening of the reads against reference genomes [17] in order to eliminate *U*. *gibba* genomic sequences, as well as other Eukaryotic organisms. The reference genomes used were: *U*. *gibba*, *Arabidopsis thaliana*, *Drosophila melanogaster*, *Cryptosporidium parvum*, *Saccharomyces cerevisae*, and *Trypanosoma brucei*. Alignments against the references were performed sequentially against the reference genomes, discarding the matching sequences in each step. We used the output from the reference genome alignments that did not match any of the references as the assembly input. The assembly was conducted using Velvet [18] using a 31-kmer threshold. The unfiltered dataset was used to estimate the overall diversity including other *Eukarya* and *Archaea* that might be discarded due to filtering.

### Diversity

Taxonomic comparison with other microbiomes, including three plant associated microbiomes with *Arabidopsis* and soybean (4562080.3, 4562079.3, 4477749.3 [6,19]; two fresh water microbiomes (4536380.3, 4441590.3 [20,21]; and two soil microbiomes (4445993.3, 4562078.3 [6,22]) as comparative external groups to establish a baseline of particular taxonomic composition within the *U*. *gibba*'s related metagenomes. For the 16S rRNA gene determination we used homology detection by means of RDP naive classifier and the Greengenes database [23,24]. We used a minimum threshold for Greengenes DB of an alignment length of 20 bases and e-value of 1e-10. Structural alignments were assembled against reference models for eukaryotes, archaea, and bacteria for the unfiltered Ugt data set with SSU-align [25]. LCA was performed using both NCBI's NR (December 2014), and the M5NR databases [26] with Megan [27].

### Annotation

Coding sequences of the Ugt and Ugm were annotated using the KEGG Automatic Annotation Server [28], using the best bi-directional hits and an e-value cut-off of 10^−10^. The filtered metagenomic assemblies were uploaded and annotated with the MG-RAST server [29]. The cut-off values used were: e-value 1e-10; minimum identity of 60%, and a minimal alignment length of 15.

### Pan-genomes fragment recruitments

Complete sequenced prokaryote genomes files were downloaded from the following URL: ftp://ftp.ncbi.nih.gov/genomes/Bacteria. A non-redundant list of genomes per species was build and used as reference to concatenate all the predicted genes of all the strain for species in a multi-fasta file. Afterwards, cd-hit-est [30] was used with a 90% identity and a word size of 8. Each non-redundant cd-hit-est output is what is called here a pan-genome, a total of 1,434 pan-genomes were calculated. Fragment recruitments were done against each pan-genome with nucmer, part of the MUMmer package [31], a minimum threshold of 0.8 in the metagenome to pan-genome alignment was used as a filter to align individual metagenomic sequence reads to the pan-genome. The pan-genome coverage is calculated as the metagenomic reads aligned against the reference pan-genome.

### Functional comparison with other metagenomes

Environmentally related metagenomes were compared using the following accessions from MG-RAST: a tropical soil from a rain forest in Puerto Rico (4446153.3, [32]) a rice rhizosphere from the Philippines (4449956.3; [33]), and a marsh, Albufera in Valencia, Spain (4516288.3; [34]). Metagenomes were selected on the basis of their potential similarities to the *U*. *gibba's* collective environment.

### Statistical analysis

All statistical analysis were conducted using R (version 3.1.2, with a X86_64 architecture). Plots were constructed using R's ggplot2, RcolorBrewer (v1.1–2), and phyloseq. Data was normalized using relative proportions, non-parametric *t*-tests were conducted to compare between groups. Multiple testing was corrected using False Discovery Rates through permutations to avoid the inclusion of false positives. For qualitative purposes the average gene abundance was used as baseline to determine the fold enrichment in Ugt. The statistical testing of diversity, and its significance were assessed with phyloseq, vegan (v.2.2–1) and metastats R packages [35–37].

### Availability of supporting data

The whole metagenome reads are available in the National Center for Biotechnology Information Short Read Archive (SRA) under accession numbers SRS941319 (Ugt), and SRS941335 (Ugm). The annotated data of this study are also available on the MG-RAST server under accessions numbers 4546121.3 (Ugt), 4546120.3 (Ugm).

## Results and Discussion

### The *Utricularia gibba* metagenomes

The sequenced trap metagenome comprised a total of 3,431,148 reads spanning 1.13 x 10^9^ bp, and that for the surrounding water plus sediment (Ugm) comprised 3,541,022 reads spanning 1.06x10^9^ bp. The GC% content was 51% ± 13 for Ugt and 58% ± 11 for Ugm. Before the assembly of the prokaryotic metagenome for both Ugt and Ugm, we filtered the raw reads against the custom eukaryotic genomic databases (see Methods). Assembly statistics are available in S1 Table. We analyzed the raw Ugt dataset to describe eukaryotic and archaea sequences within the metagenome. Almost 50% of the raw-detected rRNA genes from the Ugt sequences were assigned to eukaryotes, and by lowest common ancestor (LCA) we confirmed that most of those hits (31,373) belong to plants, with 110 hits to *Ecdysozoa* (probably a nematode), 10 matches to ciliated protozoa (*Tetrahymena*), and 129 hits to *Oomycetes* fungi (see S1 and S2 Figs). The Ugm metagenome had a total of 3,267,004 predicted CDS, and it was possible to annotate 42% of them. A total of 488,776 rRNA genes were predicted. The predicted ORFs within the Ugt metagenome comprises 3,106,769 predicted proteins of which 50.21% had a homolog in the M5NR DB. A total of 543,452 rRNA genes were identified in the Ugt.

### Estimating the *U*. *gibba* metagenome diversity

**Fig 1:**
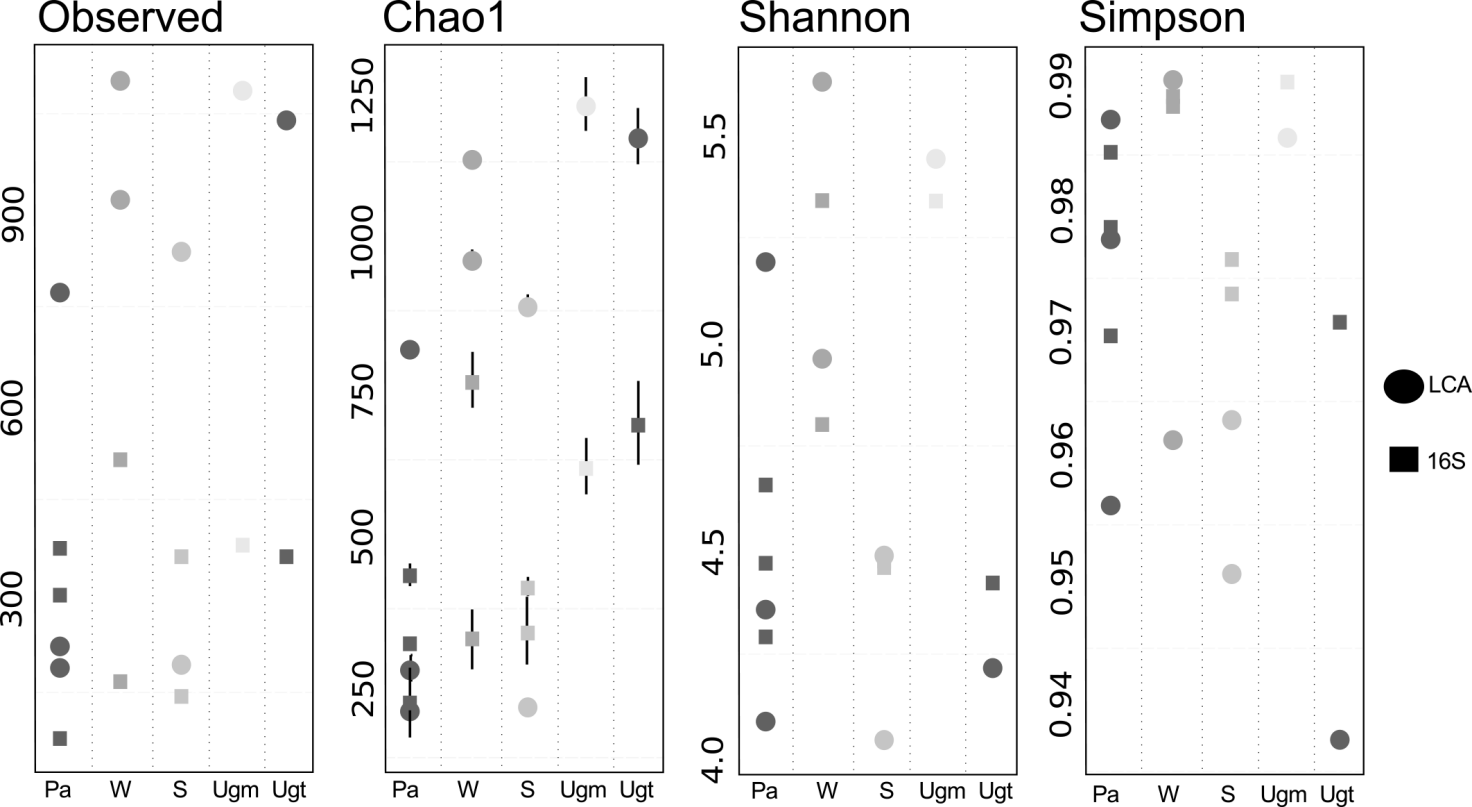
Taxonomic diversity features of *U*. *gibba* and related environments. (A) Alpha diversity measurements for *U*. *gibba’s* trap (Ugt), its surroundings (Ugm), Plant-associated (Pa), Water (W), and Soil (S) microbiomes. We made use of both 16S rRNA gene (circles) fragment assignments and lowest common ancestor (LCA, squares). Both Ugt and Ugm shows the largest expected number of species (Chao1) of the compared environments, although Ugm is more diverse (Shannon) than Ugt, and Ugt is in a less dominated environment than Ugm (Simpson).

We used three approaches to characterize the Ugt and Ugm microbiome: 16S rRNA gene profiling, the lowest common ancestor (LCA), and fragment recruitment (Fig 1). The total number of species observed with LCA was 1,087 for Ugm and 1,041 for Ugt, whereas the Chao1 index predicted a total of 1,222 species for Ugm and 1,168 for Ugt. The Chao1 index is a non-parametric estimator that considers the number of observed species and then gives an expected number of species based on the singletons (OTUs observed one time in the dataset) and doubletons (OTUs observed twice), and it has been recommended for the comparison of samples or environments with different coverages [38,39]. Shannon's index is considered to be in the range of a diverse environment for both Ugt (4.217) and Ugm (5.44). Shannon values of approximately 3–4 are considered diverse for macro-species [40], and the ranges for microorganisms vary from 0.0076 to 8.7 [41]. We calculated these values with related environments including soil, water, and plant associated microbiomes (see Methods for details about samples used for comparison), finding that their index ranged between 4.0 and 5.34, which shows that those environments are also diverse (Fig 1). Simpson's index, shows large values of dominance for both Ugt (0.9376) and Ugm (0.9865), suggesting that the two systems are not equally distributed and some species dominate these environments, but Ugt is the least dominated environment of the ones that we compared.

**Fig 2:**
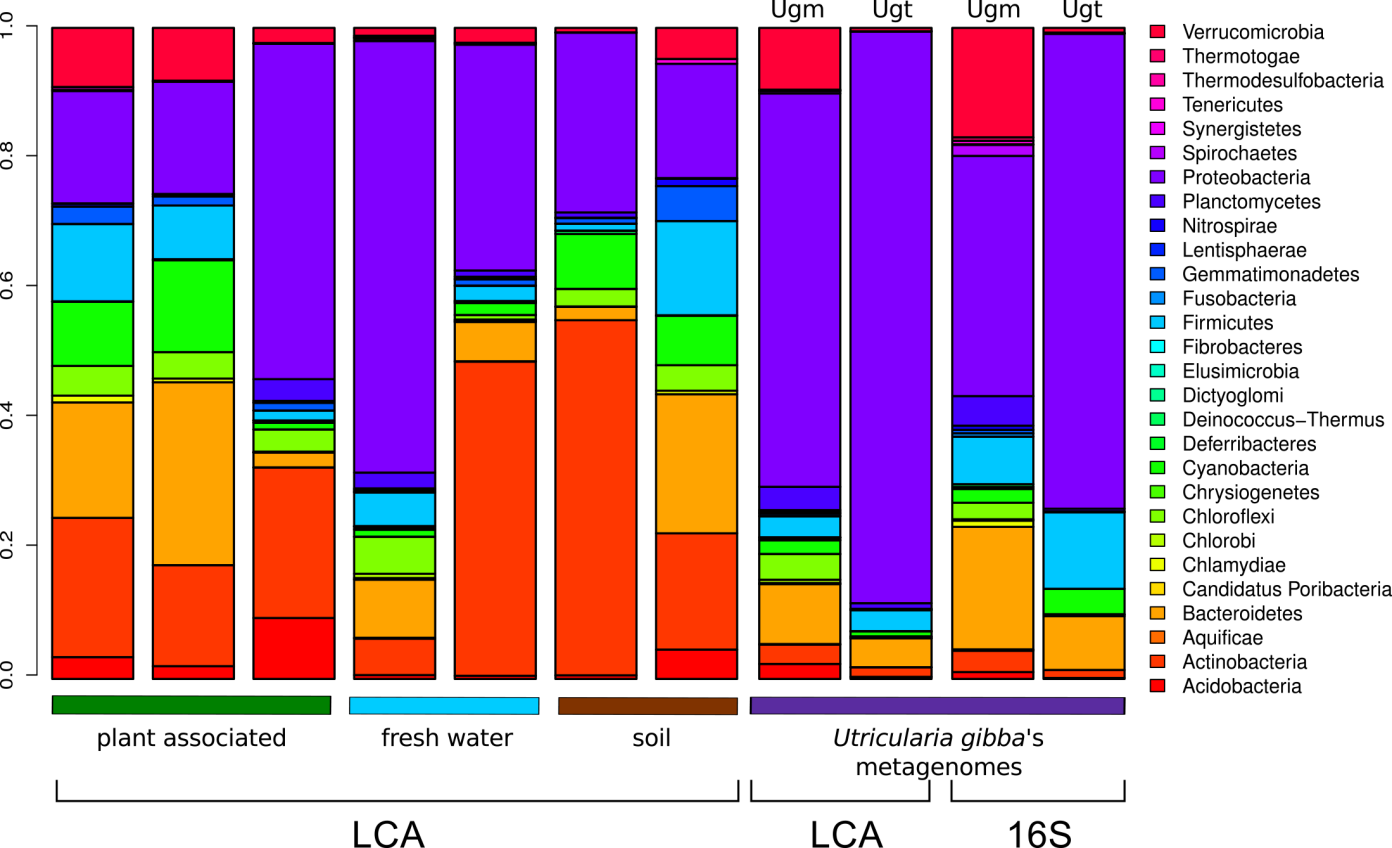
Most abundant phyla assigned for *U*. *gibba*'s trap. *U*. *gibba*'s trap (Ugt), its surroundings (Ugm), and other related microbiomes. Independently of the classification method used the most abundant phyla are: *Proteobacteria*, *Actinobacteria*, *Bacteroidetes*, *Firmicutes*, and *Cyanobacteria*.

**Fig 3:**
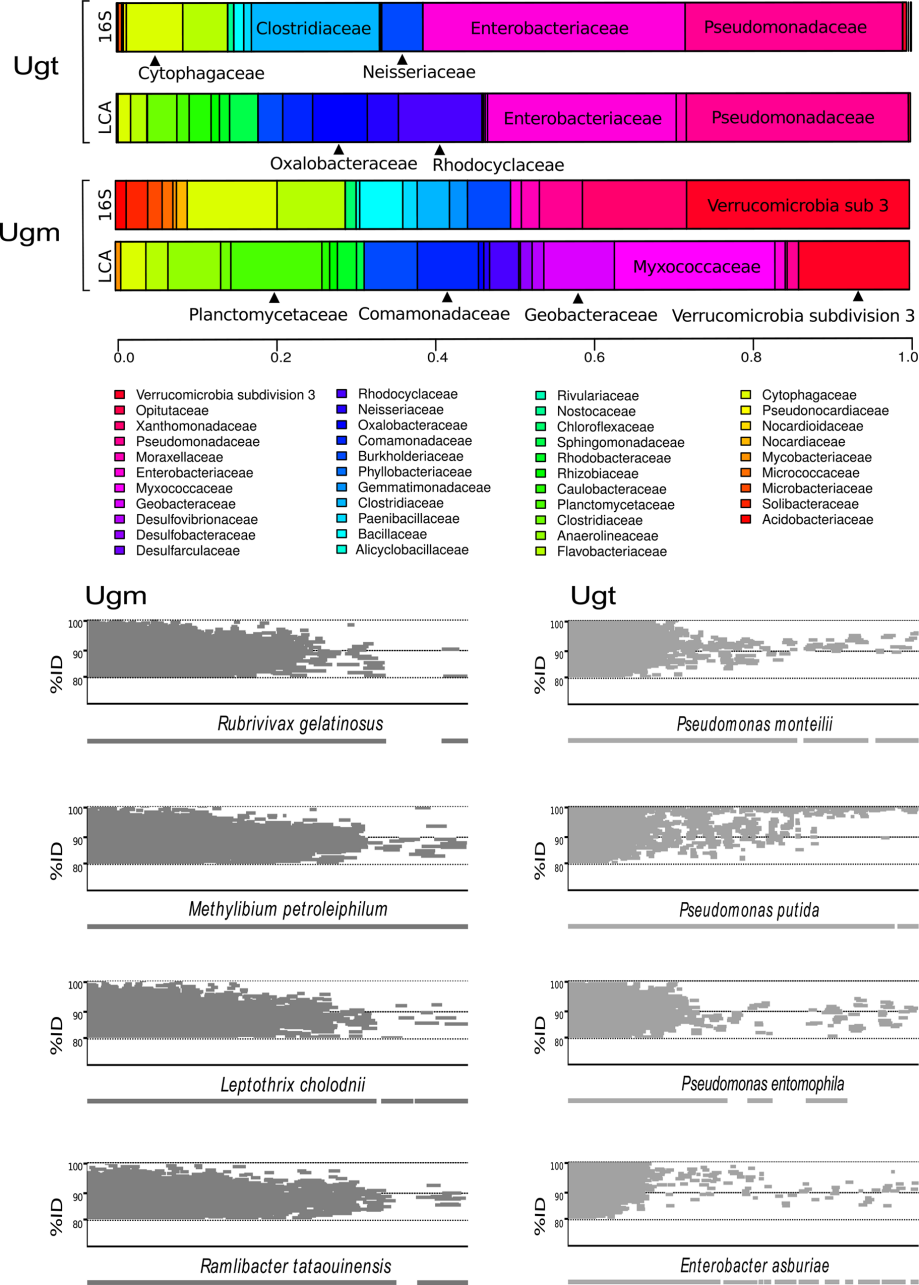
Family diversity and fragment recruitment to 1434 reference pan-genomes. The most abundant species are shown using an identity >90% of 6 frame translations for both reference and queries. In the bottom line of fragment recruitments, we can observe the consensus sequence comparing with the reference pan-genome.

Using taxonomic assignments by 16S rRNA genes, the most abundant Phyla are: *Proteobacteria* (Ugm = 36.89%, Ugt = 79.92), *Bacteroidetes* (Ugm = 18.81%, Ugt = 8.25%), and *Firmicutes* (Ugm = 7.29%, Ugt = 11.74%) (Fig 2). The relative abundances of the comparative groups for plant, water and soil-associated environments also showed that the most frequently found phyla are *Proteobacteria*, *Actinobacteria*, *Bacteroidetes*, *Firmicutes and Cyanobacteria*, independent of the classification method used. At the family level the major players in Ugm are *Myxococcaceae* (LCA = 11.15%; *delta-epsilon Proteobacteria*), followed by *Verrumicrobia* subdivision 3 (LCA = 7.7%, 16S = 9.2%; *Verrumicrobia*), *Planctomycetaceae* (LCA = 6.32%, 16S = 5.82%; *Planctomycetes*) (Fig 3). The most abundant Ugt families using both LCA and 16S rRNA gene are *Enterobacteriaceae* (LCA = 20.32%; 16S = 20.87%) and *Pseudomonaceae* (LCA = 17.29%; 16S = 23.89%) (Fig 3). The LCA method allows detection of families such as *Rhodocyclaceae* (8.99%), *Oxalobacteraceae* (5.88%), and *Neisseriaceae* (3.35%) all belonging to *Betaproteobacteria*. Some families, such as *Clostridaceae* (10.19%; Firmicutes), which is defined by its strict anaerobic capabilities were detected in Ugt by 16S rRNA gene analysis but not by LCA, albeit at a low abundance. In other systems it has been reported that *Clostridaceae* has a role in atmospheric nitrogen fixation and has been found as an endophytic bacterium in *Zea* spp. [42,43] which could explain its presence in the Ugt anoxic environment (Fig 3).

To detail the Ugt species composition we built a total of 1,434 species pan-genomes (see Methods), which were used to perform fragment recruitment analysis. The pan-genomes that recruited the most of Ugm reads (88% average identity, 0.93X coverage) corresponded to *Rubrivivax gelatinosus*, a facultative photoheterotrophic Proteobacteria usually found in freshwater, sewage, and sludge [44], followed by *Methylibium petroleiphilum*, which is capable of metabolizing Methyl ter-butyl ether (MTBE) fuel, aromatics such as benzene, and other hydrocarbons [45] (Fig 3). In the trap, the most recruited metagenomic reads were matched to *Pseudomonas monteilii* (92% average identity, 2.94X coverage). Several other Pseudomonad species were also recruited in the Ugt at a high abundance including *P*. *putida*, *P*. *entomophila*, *P*. *brassicacearum*, and *P*. *poae* (Fig 3). A complete list of recruited species, average identity and coverage is available in S2 Table.

**Fig 4:**
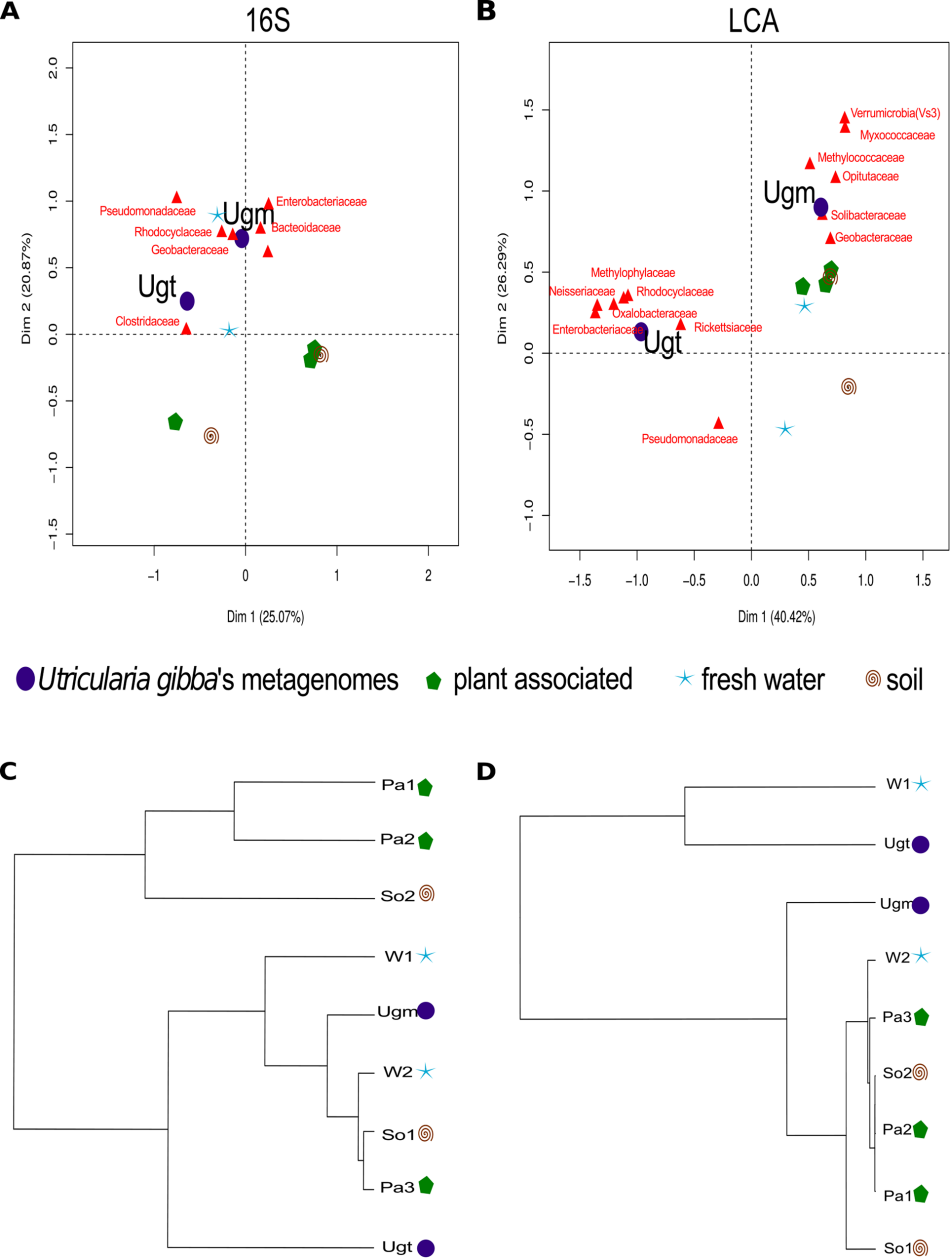
*U*. *gibba*'s traps are a unique environment according to its taxonomic profile. Correspondence analysis using both (A) 16S rRNA gene, and (B) Lowest Common Ancestor (LCA) profiles families. It can be seen that the *U*. *gibba*'s traps (Ugt) is a unique environment, this is more evident in the LCA bi-plot, where it stands on its own quadrant. This distribution explains a total of 66.71% variance. The bi-plots show the closest families to the Ugt. (C) 16S rRNA gene clustering dendrogram (complete linkage) showing the similarity between the *U*. *gibba* microbiomes and the soil, water, and plant-associated ones. The surrounding *U*. *gibba*'s environment (Ugm) is closer to water samples than it is to Ugt, and Ugt is on its own with a large branch length. Using the LCA approach a family level clustering (D) shows that Ugt and water-related samples cluster apart from all the related environments.

A bi-plot was conducted showing both families and different samples from soil, water and plant associated environments (Fig 4A and 4B). Ugt is distant from the rest of the compared environments; this is shown more dramatically by means of LCA classification, in which this arrangement explains 66.71% of the variance (Fig 4B). The families that distribute closer to Ugt are: *Enterobacteriaceae*, *Oxalobacteraceae*, *Neisseriaceae*, *Rhodocyclaaceae and Clostridaceae*, whereas Ugm has *Myxococcaceae*, *Geobacteraceae*, and *Methylococaceae* as the most abundant families. Sample similarities were also inspected by cluster analysis, and in both 16S and LCA ordering plant-associated and soil samples tend to cluster together, while Ugt is always a sister group to water samples, as is the case for Ugm (Fig 4C and 4D). Fine details of the family abundances across all samples are available as heat-maps (S3 and S4 Figs).

Recently, the matagenome from some other species of carnivorous plants have been sequenced, including *Genlisea aurea*, and 4 other *Utricularia* species [14,15]. The *Genlisea* metatranscriptome work defines diversity at the genus level (39–188 genera) and define the “active” microbiome as a function of the relative abundance (>0.1%) of transcripts. In a meta-analysis using representative hits of 16S rRNA gene Ugt is more diverse than the *Genlisea* traps (188 phylotypes at the genera level), the Ugt hosts 398 bacteria, 2 *Archaea*, and 797 eukaryotic genera, the complete Ugt genera list and its details are available as S3 Table. The dominance of *Proteobacteria* is common to both *Genlisea* and Ugt, as well as other plants. Although, *Pseudomonas* is the most abundant genus in Ugt while *in Genlisea* the genera *Asaia* and other *Rhizobiales* are enriched resembling a traditional root microbiome in the case of *Genlisea* [15].

The metatranscriptomic and microbiome analysis in the other *Utricularia* species focused on diazotrophic diversity [14]. In this work we collected similar taxonomic diversity to the one described previously in other *Utricularia* species, even in different continents. Going further, we were able to identify up to species pan-genomic level in Ugt and reconstruct their genomes with fragment recruitments analysis. We found that *Pseudomonas monteilii* is the most abundant recruited species, which had been found, along with other *Pseudomonas* species, to be a plant growth promoting bacteria [46,47], and is in agreement with the previous findings of the presence of *Pseudomonas* species in sister *Utricularia* species [14].

### Functional diversity in the traps, its surroundings, and environmental related metagenomes

**Fig 5:**
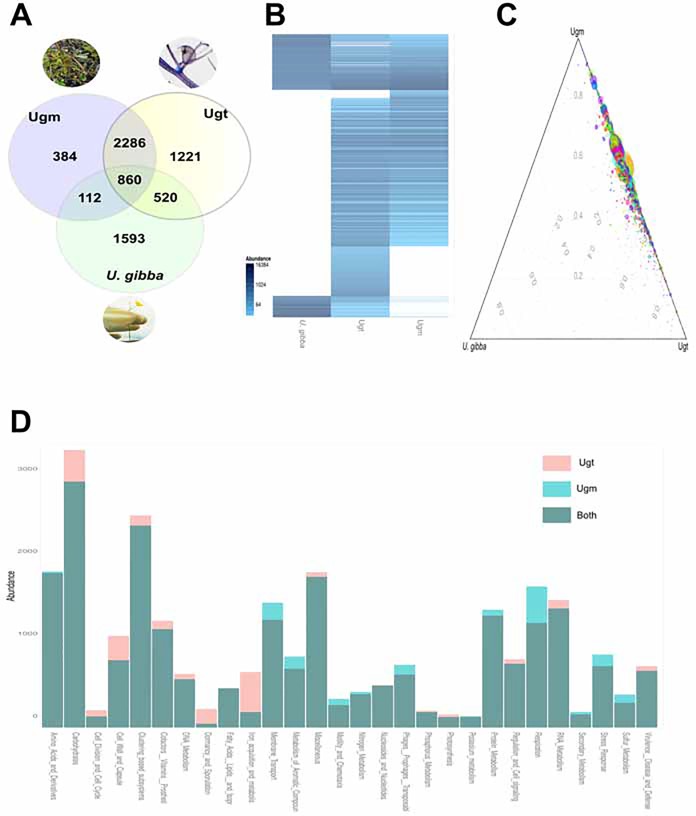
Comparative functional genomics and the complement between *U*. *gibba*'s genome, and its trap (Ugt). (A) Venn diagram comparing the amount of shared genes between the plant's genome, Ugt, and Ugm (using KEGG orthologs). (B) A Heat-map showing the overall metabolic complement supplied by Ugt, note that most of this complement is shared with Ugm and only a subset is in greater abundance in Ugt. (C) A ternary plot in which each dot corresponds to a KEGG ortholog and its diameter is proportional to its abundance. This plot shows that most of the predicted gene functions are shared between Ugt and Ugm, whereas a minimal portion of genes are shared directly between Ugt, Ugm and the plant's genome. (D) Major functions coded by both *U*. *gibba*'s trap (Ugt) and its medium (Ugm). The categories used correspond to level 1 of the SEED's hierarchy.

A comparison between the *U*. *gibba*'s predicted proteome against Ugt and Ugm was conducted by means of KEGG orthology (see Methods). These comparisons allowed us to investigate enzymes and general metabolism proteins shared among the plant, its trap and the surroundings (S4 Table). The core of the proteins in Ugt, Ugm and the plant is reduced to 860 shared proteins (Fig 5A). Most of the shared genes are between Ugt and Ugm (3146), thus supporting Ugt as a subset of Ugm. Ugt shows unique features (N = 1,221) which could be part of the microbiome's functional input to its host (Fig 5A). The shared and unique features for Ugt, Ugm and *U*. *gibba* are easily visualized by means of ternary and heat-map plots (Fig 5B and 5C). When comparing the functional diversity of Ugt and Ugm at the SEED's higher hierarchy level (Fig 5D) the overall picture indicates that Ugt metagenome encodes genes for carbohydrates metabolism (Ugt = 3,148; Ugm = 2,789), iron acquisition and metabolism (Ugt = 621, Ugm = 169), and dormancy and sporulation (Ugt = 201; Ugm = 37). By contrast Ugm devotes a larger number of genes to respiration (Ugm = 1,596; Ugt = 1,180) and stress response genes than Ugt (Ugm = 822; Ugt = 689) (Fig 5D). These results support the environmental niche of Ugt and suggest that the microbes that colonize the trap take advantage of the carbon sources provided by the plant and that iron is an active limitation for both the plant and its microbiome. The dormancy and sporulation category probably reflects the bias for organisms like *Clostridaceae* while not being the dominant species within the trap, its presence in this niche cannot be neglected because O_2_ limitation inside the trap selects for anaerobic and facultative aerobic bacteria.

**Fig 6:**
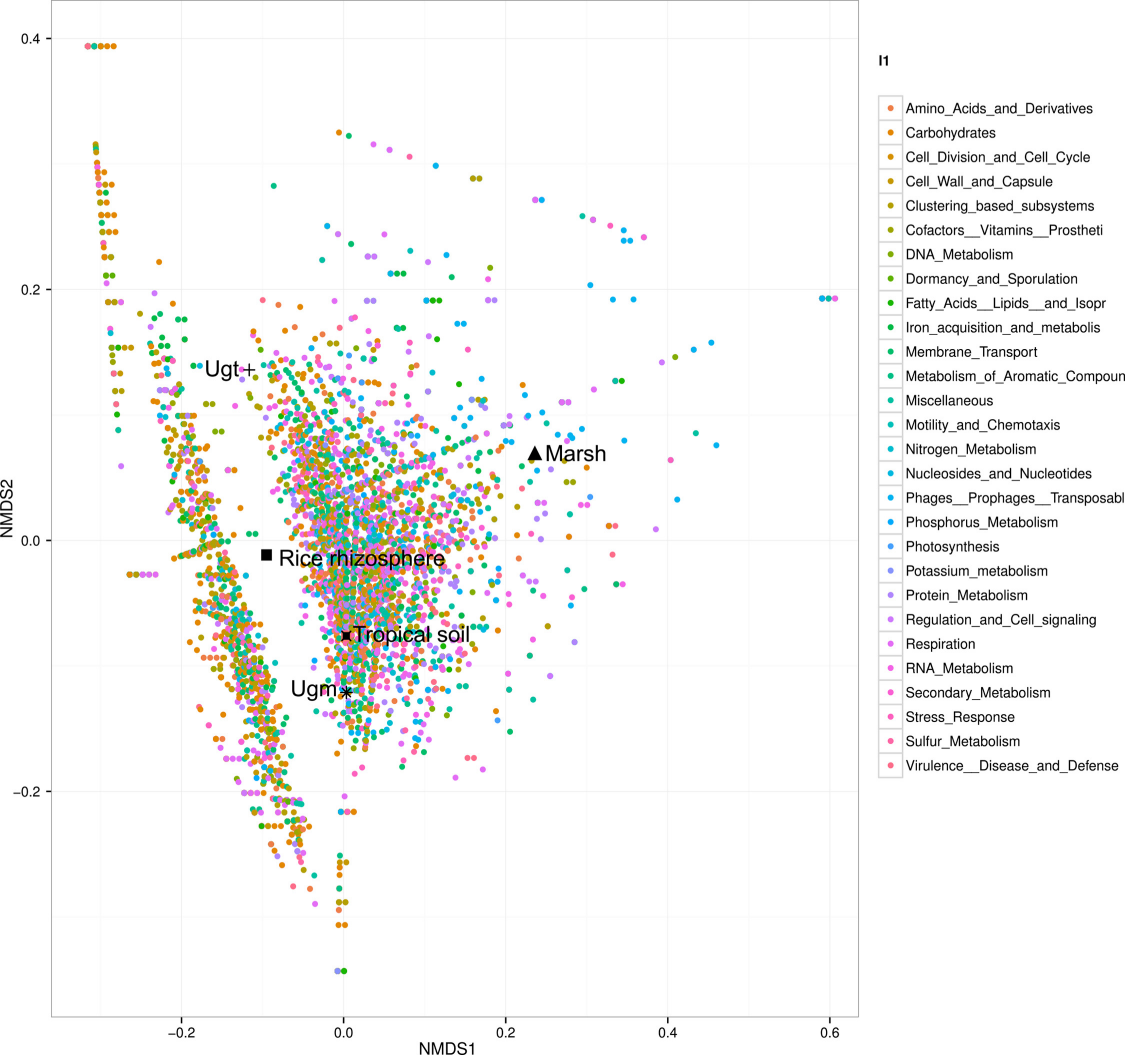
The Ugt metagenome is quite unique, even with the predicted functional analysis. A Non-Metric Dimensional Scaling (NMDS) bi-plot analysis is shown and using 11,891 annotated features from M5NR DB, where level 1 SEED's functional hierarchy is used to color the predicted genes. The analysis confirms the uniqueness of Ugt with other metagenomes, and shows that Ugm is functionally clustered apart from Ugm.

The selected metagenomes for the environmental comparisons were from related environments such as a rice rhizosphere, a tropical soil, and a marsh (see Methods). Nonmetric Multidimensional Scaling (NMDS) functional analysis confirmed that the taxonomic Ugt uniqueness, a total of 11,891 annotated features were used across all samples, placing the Ugt cluster apart (Fig 6). The annotation, detected significant Ugt over-represented (OR) genes with its false discovery rate corrected p-value, and the 250 most abundant genes within Ugt are available in S5 Table.

The anaerobic conditions within the trap were supported by the presence of genes such as *arcB* which is an aerobic respiration control sensor, a nitrate/nitrite sensor histidine kinase NarQ (14-fold), *ntrY* (6-fold) which is a nitrogen regulation sensor, the nitric oxide deoxygenase (33-fold), and NirD (18-fold) which is part of the NirBD complex that reduces NO_2_ to NH_3_ [48]. The presence of MazG (11-fold) could be explained in several ways, for example that is the result of NTPs hydrolysis because of ROS damage, or as a sensing system for amino acid starvation [49].

A putrescine importer (*puuP*, 10-fold) and its repressor (*puuR*, 8-fold), as well as the putrescine binding protein (*potF*, 5-fold), and its exporter PotE are present in Ugt, suggesting the contribution of polyamines to the host by Ugt microbes. It has been shown that *puuP* is an essential gene when only putrescine is used as Nitrogen/Carbon source [50].

Nitrogen plays an important role in the Ugt microbiome. First, the Ugt microbiome has genes encoding for metabolism of cyanophycin (p = 0.00674), which is a reserve material for C and N, and is also present in the specialized cyanobacteria heterocists where nitrogen fixation takes place [51]. Several significant Over Represented (OR) nitrogen related proteins such as NifE (p = 0.00009), and NirN (p = 0.0239) are included in Ugt. NifE codes for an assembly protein for a nitrogenase with a FeMo-cofactor and because of the limited Oxygen within Ugt it makes sense to have the Nitrogen fixing capabilities. NirN, by contrast is a largely ubiquitous gene among denitrifying bacteria that encodes a dehydrogenase involved in denitrification carrying out the last step of heme d1 biosynthesis [52]. The observation of this in *Pseudomonas* strains corresponds with our results from fragment recruitments. The energy production via denitrification is relevant when growing under anaerobic conditions such as those present in the Ugt. The possible role of the Ugt microbiome in the urea cycle and the polyamines is reinforced by an ORF for putrescine utilization pathways (p = 0.005), cyanophycin related proteins (p = 0.007), and arginine and ornithine degradation enzymes (p = 0.019). This is also supported by the presence of Glutathionylspermidine amidohydrolase (p = 0.018), which is a bifunctional enzyme with synthetase and hydrolase domains that could help to regulate antioxidant stress in the trap environment [53].

Phosphorus uptake and assimilation in Ugt are represented by genes encoding the phosphatases PldB (8-fold), and PldA (7-fold). Here, in comparison with other metagenomes we found that the master transcriptional regulator, responsible for the sensing and response to Pi from the environment, PhoB [54], is significantly OR in Ugt (p = 0.4251). Another piece of evidence for the relevance of phosphonate scavenging is the finding that 2-aminoethylphosphonate pyruvate aminotransferase is OR in Ugt (p = 0.0367), as is an alkylphosphonate ABC transporter (p = 0.0054); it is also supported by a large set of the *phn* related genes are over-represented in the Ugt (*phnCDIJKP*).

Within the unique features of Ugt, which were not present in the other environmental metagenomes, we found several proteins that could help bacteria to deal with the trap's harsh environment via osmoprotection such as K00697 coding for OtsA, a trehalose phosphate synthase; K05845-46 with the genes *opuC* (4.6-fold), *opuBC* (13-fold) and *proP* (10-fold) which are part of the osmoprotectant transport system; and a range of two component system coding genes such as *envZ* (20.5-fold) which works an osmolarity sensor. It has been reported that the Ugt maintaining negative pressure within the trap, and also that water is removed within Ugt when a prey is captured, thus making it necessary to have strategies cope with dehydration and preserve the cell functioning [55]. TauB (5.5-fold) is present and its part of an ABC transporter, and taurine could help to cope with ROS, via NADPH oxidase [56]. Along these lines, glutathione related proteins are found to be OR in Ugt: reductases (7.8-fold), transferases (4.4-fold), and *ketFG* (7_fold; glutathione-related potassium efflux system). Another osmoprotection protein, GbcA, a glycine betaine demethylase is only in Ugt.

There are functional similarities between Ugt and other analogous systems like the Rice endophytes. In the rice's endophytes metagenome [7], there are several ROS detoxification predicted proteins which are in line with the findings for Ugt. It has been proposed previously that endophytes require ROS detoxification enzymes to successfully colonize the plant, this has been proposed for two of the species of plant growth-promoting bacteria found in the trap as *Enterococcus* sp. and *Klebsiella* sp. *[57,58].*

Some DNA repair and recombination coding genes are OR in Ugt, as is the case for the DNA repair protein RAD51 (p = 0.276) which is a eukaryote homologue to RecA and works in homologous recombination [59], and the formamidoyrimidine-DNA glycosylase (Fpg; p = 0.277) which is a DNA repair enzyme that removes oxidized purines [60].

The microbiome of *U*. *gibba* is probably contributes to cell detoxification within the trap, as well as resistance to some antibiotics, as is the case for the multidrug efflux transporters MexB (p = 0.04466), and MexD (p = 0.00748). MexB is part of the *mexA-mexB-oprK* operon, and its outer membrane is OprK; these genes account for the pyoverdine export, and resistance to β-lactams [61]. By contrast, MexD is part of the efflux system MexC-MexD-OprJ which gives resistance to fourth generation antibiotics, and fluorine-quinolones but not to regular β-lactams [62]. The production of antibiotics could influence the Ugt microbiome composition; for example, in the particular case of the bacteriocin TldD (p = 0.01223), which acts as a protease and its required to maturate the mycrocin B17 prior to its export, a mutation in *tldD* results in failure to export the mycrocin [63]. The community structuring via antibiotics and bacteriocins has been found in other environments, and it occurs between closely related species, suggesting that the selection struggle occurs even at a narrow phylogenetic distance [64,65].

Iron scavenging strategies and metal homeostasis are well represented in Ugt by means of the iron complex receptor, permease, and substrate binding proteins (K02014-16, K16089). The proteins responsible for metal homeostasis are also OR in Ugt with *cusS/copS* (22.5-fold) which is a heavy metal sensor, a nickel/cobalt exporter (*rcnA*, 11-fold), zinc transport protein (6-fold), and a Cu(I)/Ag(I) efflux membrane protein (5.25-fold). An interesting function detected only on the Ugt metagenome, is carried out by NfuA a nickel-sulfur protein which allows the use DNA as a sole carbon source [66]. The enrichment of iron scavenging mechanisms have been seen also in the rice endophytes [7], and it has been suggested that this is a potential of the endophyte communities to over-compete pathogens, this has been observed with some cultured endophytes [67].

Regarding the hydrolytic contribution of the Ugt microbiome to its host, there are a plethora of peptidases, proteases, and hydrolases that are much more abundant in Ugt than Ugm (complete list is available as S4 Table). From the *U*. *gibba*'s transcriptome we have learned about the role of the plant in the hydrolytic activity within the traps, which was thought to be determined solely on is microbial community [9], but the coding potential in the Ugt microbiome for hydrolytic activities certainly have the capacity to complements the *U*. *gibba* encoded hydrolytic enzymes.

A qualitative analysis of the 250 most-abundant proteins for all the compared metagenomes is shown in S5 Fig, and the data are available in S5 Table. The general overview of the qualitative analysis is that Ugt is merely a subset of Ugm but with differential OR features supporting the Ugt uniqueness. One of the most abundant Ugt predicted proteins is siderophore related, which is specifically involved in the pyoverdine synthesis (S5 Fig Ids = 14964, 14968), and is well known to be a *Pseudomonas'* trait [68]. *T*he presence of 2-oxoglutarate dehydrogenase (S5 Fig id = 8840) which is highly abundant in Ugt, may contribute to a plant's ammonium input [69]. The presence of the 2-oxoglutarate dehydrogenase (OGDH), and several genes associated with the biosynthesis of its cofactor thiamin diphosphate (*thiL*, *thiE*, *thiB* and *tbpA*), along with the enzyme aconitase, which was found to be significantly abundant in Ugt (p = 0.0238), support a reverse TCA cycle, replacing the carbon source for glutamine with the synthesis of acetyl CoA, citrate, and fatty acids under anaerobic conditions [70,71]. Nitrogen assimilation via glutamine (GS) and glutamate synthetase (GOCAT) has been suggested to be a metabolic signal regulating both nitrogen and carbon metabolisms, this requires 2-oxoglutarate as a main source [69].

Regarding to previous *Genlisea* and *Utricularia'*s metatranscriptomes this work enriches the knowledge in carnivorous plant microbiomes. When defining the active microbial population metatranscriptomics is a choice, but we are also aware that metagenome sequencing allows describing the taxonomic and metabolic community structure, which is necessary to later distinguish between the active and resilient inhabitants from the transient ones. Although, we are aware that some species and genes described in this work may not be transcriptionally active, we describe the most represented taxa and genes in the Ugt microbiome, without PCR amplification biases. The metatranscriptomic approach poses some biases generated by rRNA depletion/mRNA enrichments treatments and caution must be taken because of the labile life of mRNA, some transcripts are so specific that are present only during seconds to over an hour and then further degraded [72,73]. Finally, the goal of describing a metagenome is different from a metatranscriptome, the metagenome stands for describing the composition and the metatranscriptome goal is to study gene regulation. Moreover, it has been observed that 41% of transcripts of a metatranscriptome does not show significant differences when comparing the gene abundances against the reference metagenome [74].

Finally, the connection between the taxonomic and metabolic diversity is congruent in the Ugt. The metabolic repertoire of the *Pseudomonas* genus is vast, and in the case of the particular species that recruited the most Ugt reads, *P*. *monteilii* has a wide range of catabolic capabilities, including hydrocarbon degradation of benzene, toluene, ethyl-benzene [75], formaldehyde [46], and organophosphate compounds [47]. Although *P*. *monteilii* and other well represented species in Ugt such as *Klebsiella variicola*, and *Enterobacter* sp. R4-368 were first isolated from clinical samples and treated as potential human pathogens [76], they also have been shown to be nitrogen fixers when living as plant-associated bacteria [77,78] and providing other plant benefits like phosphorus mobilization, fungi pathogen inhibition, and providing plant hormones to its host [79–81]. Previous reports on the model *Arabidopsis* have highlighted the thin line between being a pathogen or a beneficial bacterium. For instance, tiny changes, such as modifications to a *quorum sensing* system in *Pseudomonas aeruginosa*, could switch it from human pathogen to be a plant growth-promoting bacteria [82], and further work on *U*. *gibba*'s microbiome could test this duality.

## Conclusions

The *U*. *gibba*'s trap microbiome is a unique, and highly diverse bacterial community, with particular species composition even when compared with its surrounding environment. The *U*. *gibba*'s trap metagenome helped to find mechanisms by which microorganisms could help the plant host to cope with the harsh trap environment with plentiful of ROS and its mutagen effects, some of these mechanisms are shared with analogous systems like rice. This study complements the previous *U*. *gibba* transcriptomic study, and its whole-genome sequencing, by showing the metagenome complement supplied by its microbial inhabitants. At the pan-genomic level, several *Pseudomonas* species seem to be major players within the trap along with several species like *Klebsiella* and *Enterobacter*, and we were able to map the metagenomic reads to reference pan-genomes, and giving the chance for further environmental genomes assemblies The predominance of bacteria in the trap that have been previously reported to be plant growth promoters could suggest that in *U*. *gibba*, a rootless plant, the microbiome needed by the plant to enhance its capacity for nutrient uptake and assimilation, and healthy development has switched from being associated with the root system to being associated with the trap. This work also opens new venues regarding directed isolation of the most abundant bacteria within the trap and test their metabolic capabilities, as well as testing their temporal resilience to determine whether the trap microbiome is stable or highly dynamic depending on seasonal or geographic conditions. The metabolic resemblances with other species like the rice, and the taxonomic conservation compared with other *Utricularia* species are larger than chance and opens the quest for a metabolic core of genes and species in the endophyte microbiome of aquatic insectivorous plants.

## Supporting Information

S1 Fig The Lowest Common Ancestor profile for the raw Ugt data set, the size of the circle is proportional to the number of sequences assigned. (PDF)

S2 Fig Structural 16S rRNA profiles for *Archaea*, *Eukarya*, and *Bacteria* for the unfiltered Ugt data set. (PDF)

S3 Fig Heat-map showing the 16S rRNA profiles for Ugt, Ugm and the comparative microbiomes. (PDF)

S4 Fig Heat-map showing LCA profiles for Ugt, Ugm and the comparative microbiomes. (PDF)

S5 Fig Heat-map showing the top 250 functions represented for Ugt and the compared metagenomes. This ordination is useful for making a qualitative analysis and describing the major features of Ugt as compared with Ugm, soil, rice rhizosphere and a bog metagenome.
(PDF)

S1 Table Metagenomes assembly statistics. (XLS)

S2 Table Pan-genome recruitments for both Ugt and Ugm. It shows the reference pan-genome, its query length, coverage, and average identity of the overall alignment.
(XLS)

S3 Table Taxonomy summary of best hits in the M5RNA DB for Ugt. (XLS)

S4 Table Kegg Ortholog annotation for *U*. *gibba*, Ugt, and Ugm. (XLS)

S5 Table Complete data set annotation for Ugt, Ugm and the compared metagenomes. P-values, means of significant OR features for Ugt, and the complete information for the qualitative analyses shown (fold change).
(XLS)
