# Supplementary figures and images for: The Metagenome of Utricularia gibba's Traps: Into the Microbial Input to a Carnivorous Plant
Source: PLoS One. 2016 Feb 9;11(2):e0148979. doi: 10.1371/journal.pone.0148979 (PMC4747601; doi:10.1371/journal.pone.0148979)

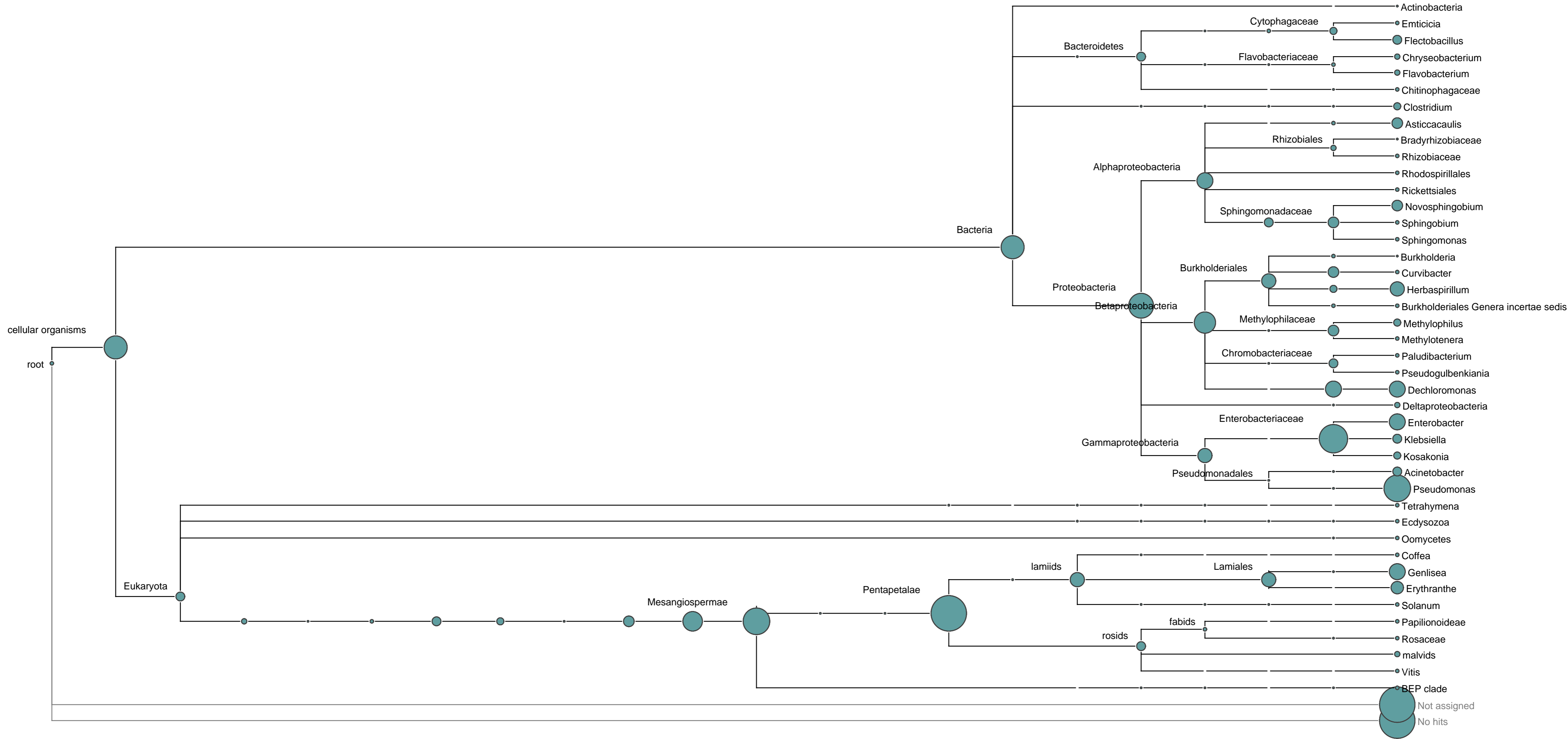

Supplement: S1 Fig — (PDF) [file pone.0148979.s001.pdf]

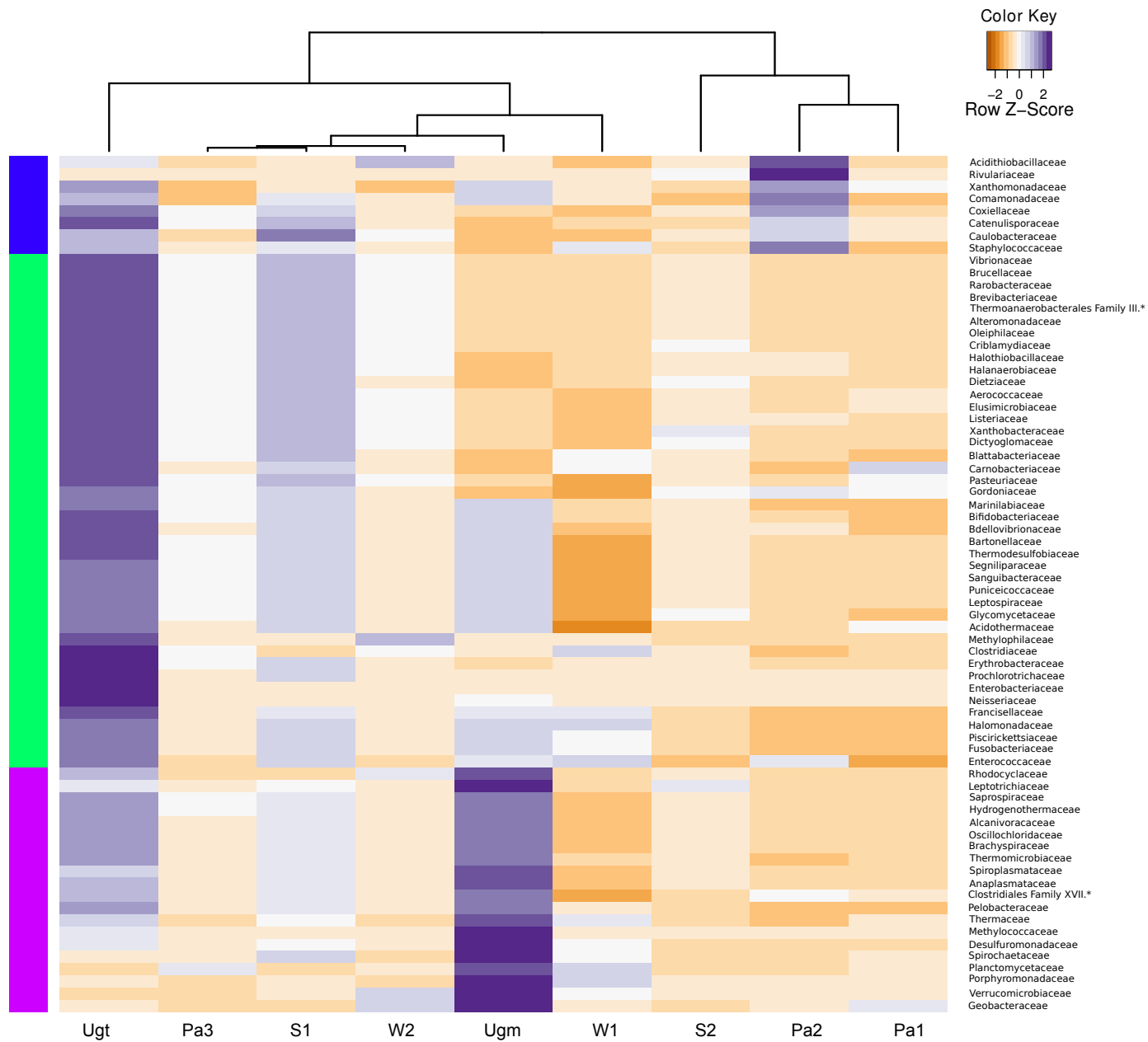

Supplement: S3 Fig — (PDF) [file pone.0148979.s003.pdf]

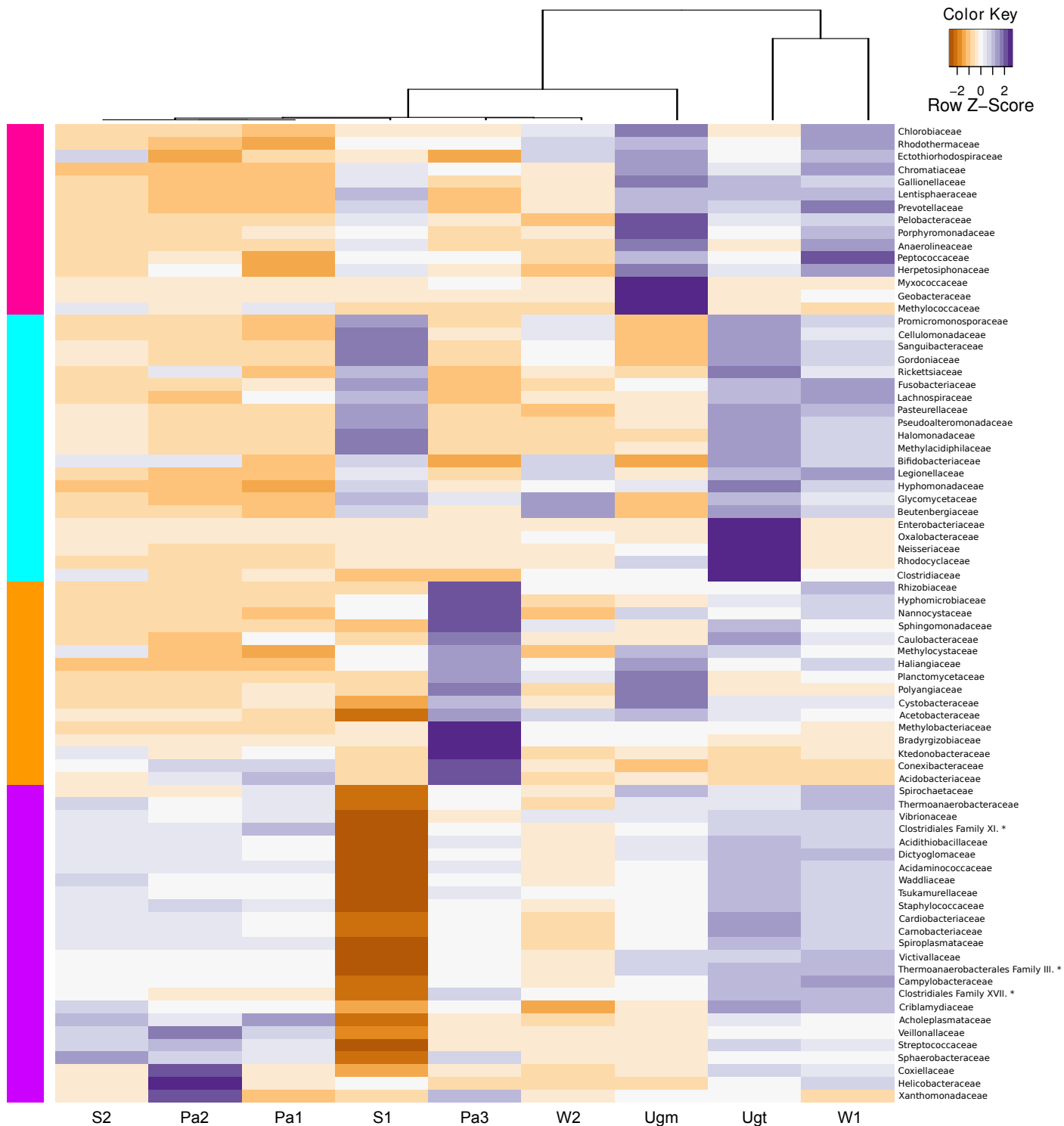

Supplement: S4 Fig — (PDF) [file pone.0148979.s004.pdf]

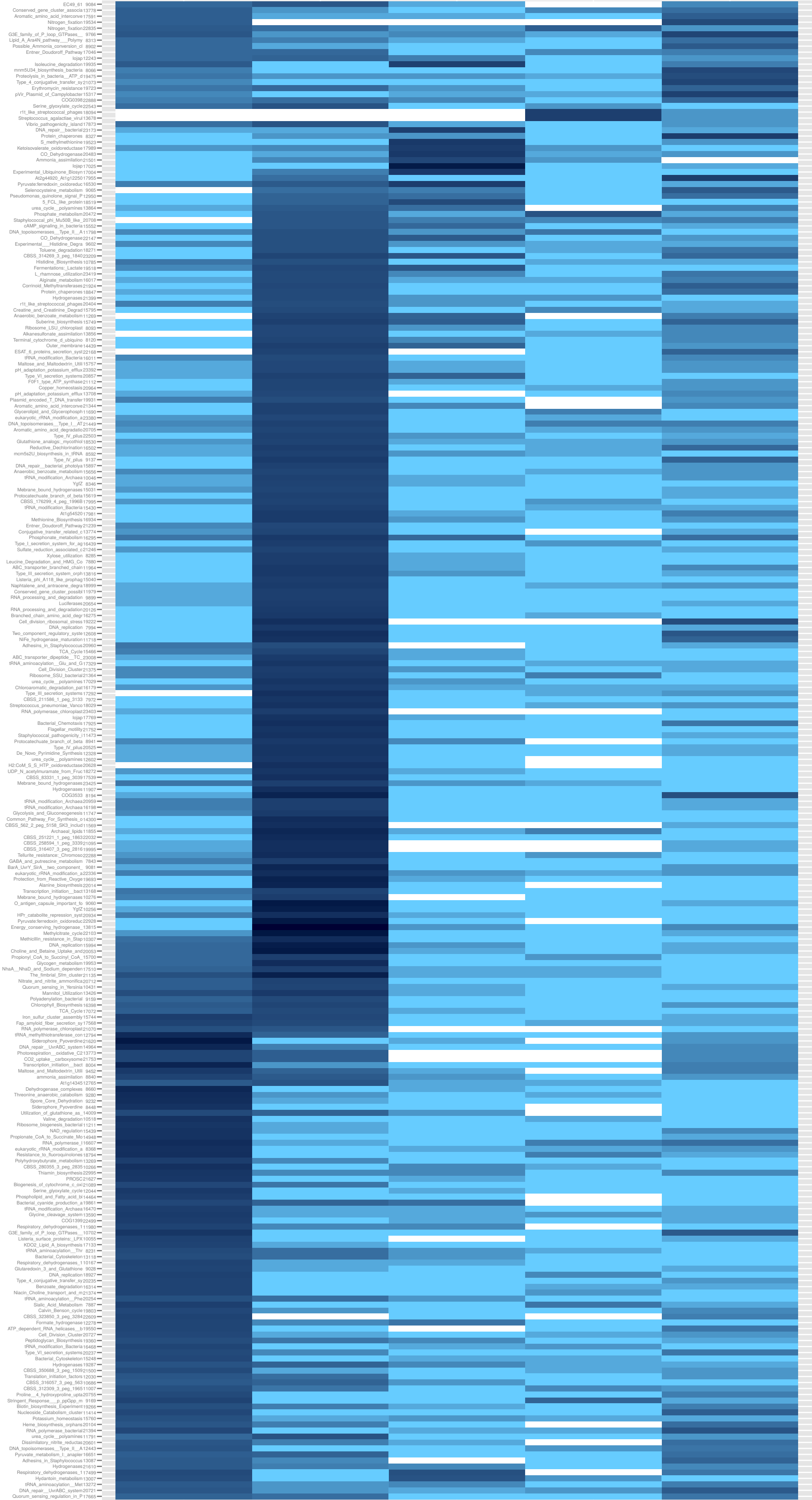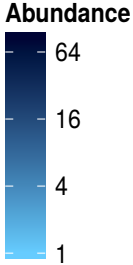

Supplement: S5 Fig — This ordination is useful for making a qualitative analysis and describing the major features of Ugt as compared with Ugm, soil, rice rhizosphere and a bog metagenome. (PDF) [file pone.0148979.s005.pdf]
