# Supplementary material for: The Metagenome of Utricularia gibba's Traps: Into the Microbial Input to a Carnivorous Plant
Source: PLoS One. 2016 Feb 9;11(2):e0148979. doi: 10.1371/journal.pone.0148979 (PMC4747601; doi:10.1371/journal.pone.0148979)
Supplement: S2 Fig — (PDF) [file pone.0148979.s002.pdf]

model	#pos	#bps	#seqs	description
archaea	1508	471	23	information content per position

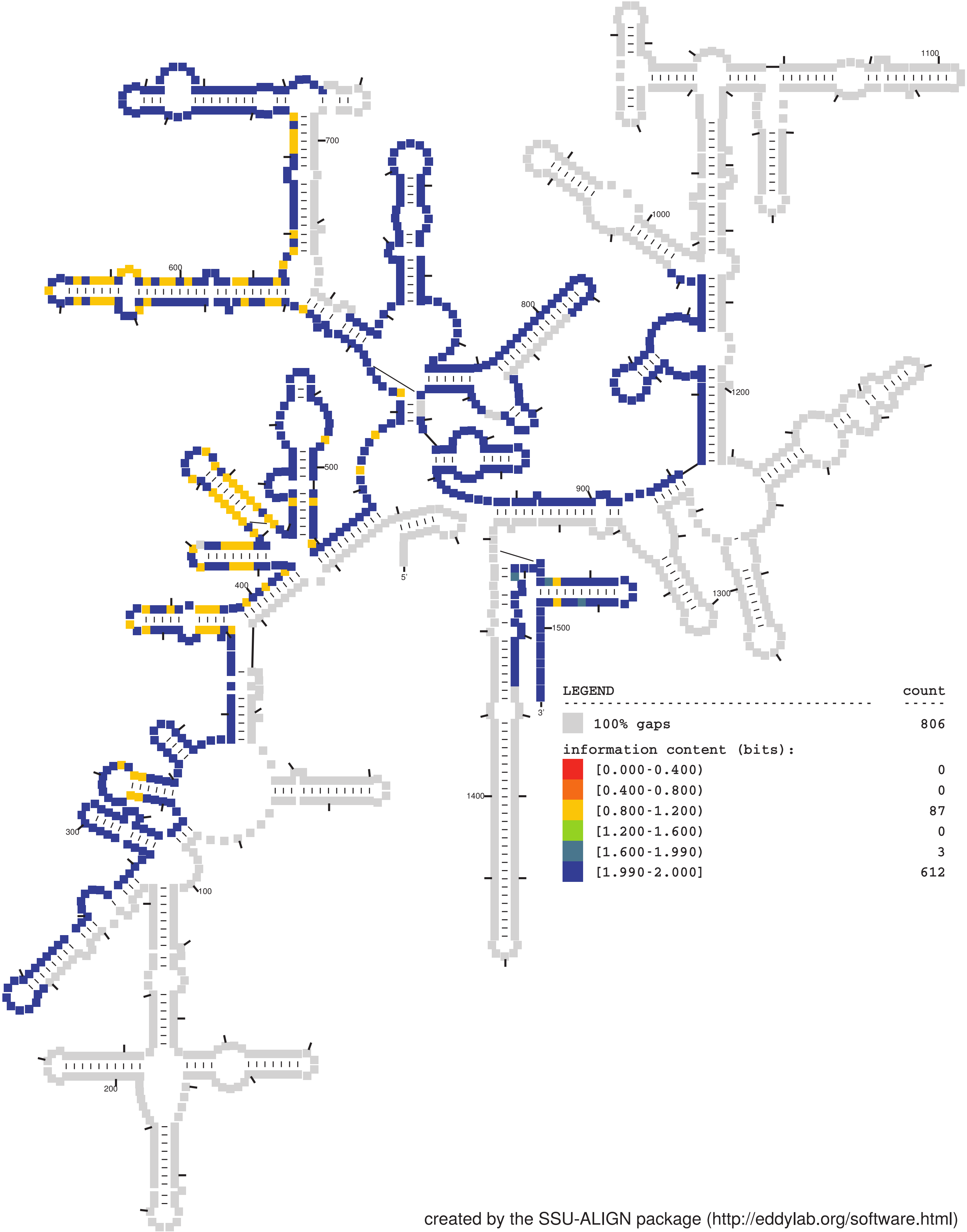

LEGEND	count
100% gaps	806
information content (bits):	
[0.000-0.400)	0
[0.400-0.800)	0
[0.800-1.200)	87
[1.200-1.600)	0
[1.600-1.990)	3
[1.990-2.000]	612

created by the SSU-ALIGN package (http://eddylab.org/software.html)
structure diagram derived from CRW database (http://www.rna.ccbb.utexas.edu/)

alignment file: trampa-ssout/trampa-ssout.archaea.stk

model	#pos	#bps	#seqs	description
bacteria	1582	480	6313	information content per position

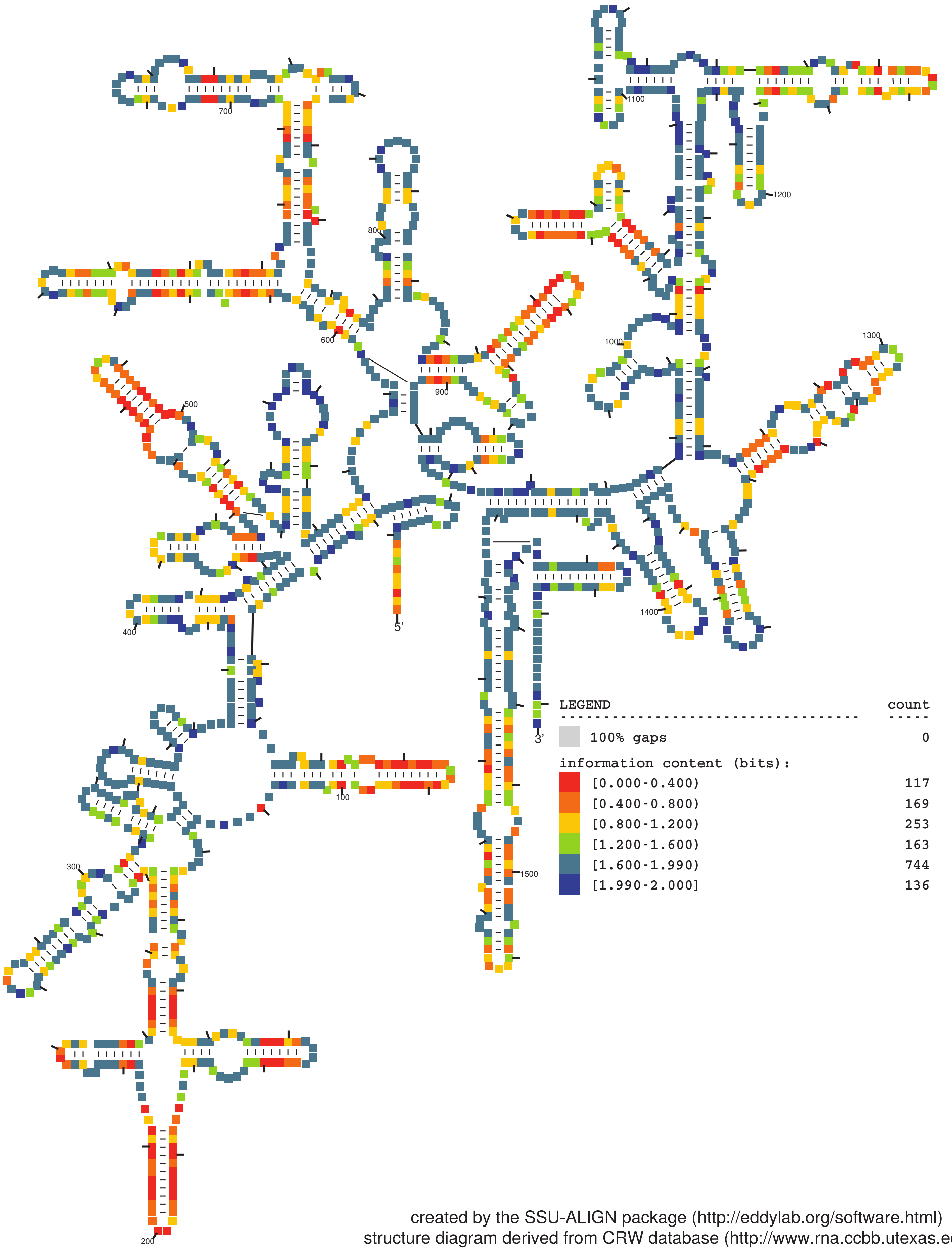

created by the SSU-ALIGN package (http://eddylab.org/software.html)
structure diagram derived from CRW database (http://www.rna.ccbb.utexas.edu/)

alignment file: trampa-ssout/trampa-ssout.bacteria.stk

model	#pos	#bps	#seqs	description
eukarya	1881	448	6328	information content per position

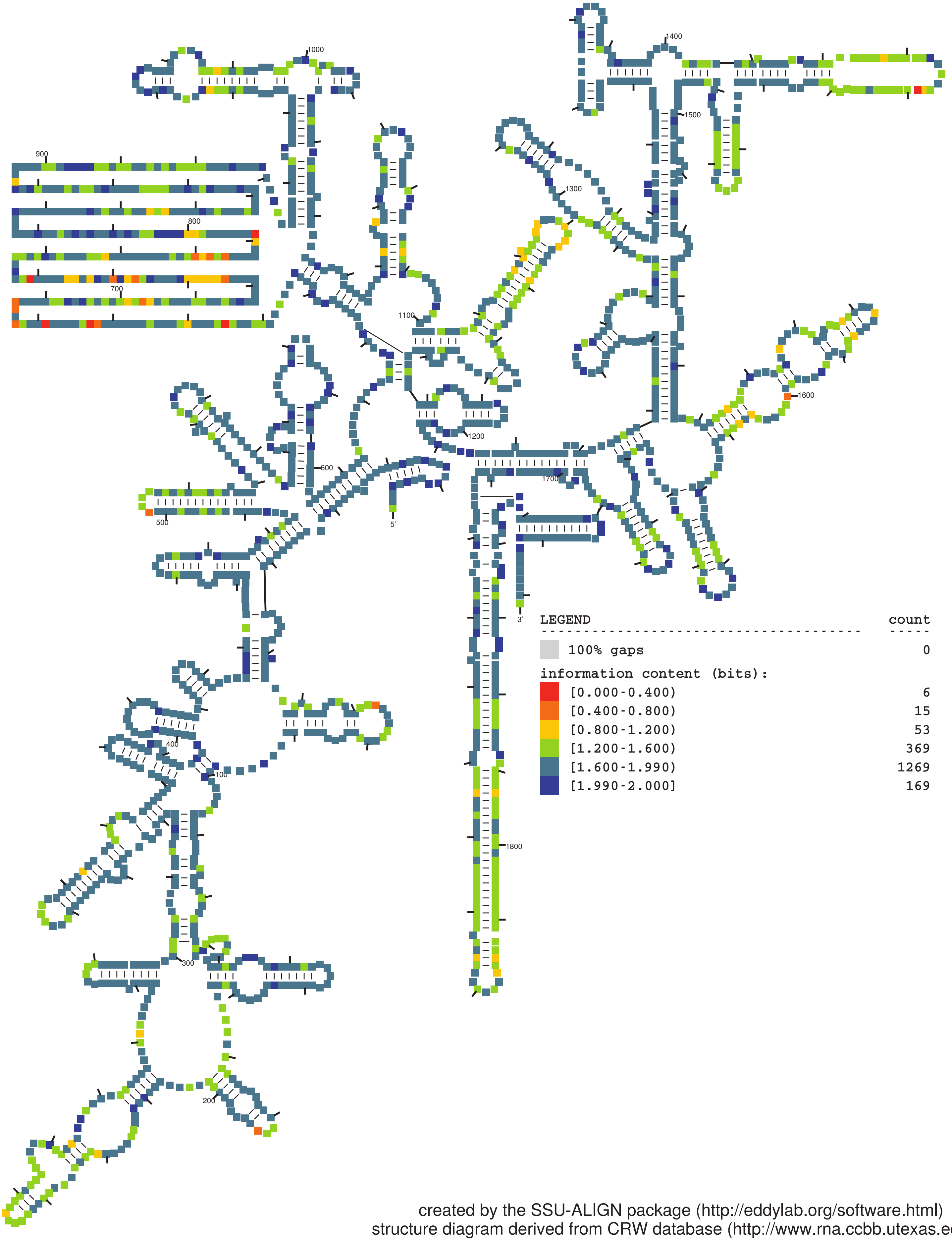

LEGEND	count
100% gaps	0
information content (bits):	
[0.000-0.400)	6
[0.400-0.800)	15
[0.800-1.200)	53
[1.200-1.600)	369
[1.600-1.990)	1269
[1.990-2.000]	169

created by the SSU-ALIGN package (http://eddylab.org/software.html)
structure diagram derived from CRW database (http://www.rna.ccbb.utexas.edu/)

alignment file: trampa-ssout/trampa-ssout.eukarya.stk
